# Elucidating the molecular genetic basis of cluster headache: delineation of the genetic architecture by exome sequencing

**DOI:** 10.1186/1129-2377-14-S1-P34

**Published:** 2013-02-21

**Authors:** L Southgate, S Scollen, W He, A Moss, MA Simpson, B Zhang, L Xi, T Schlitt, ME Weale, CL Hyde, JC Stephens, C Sjöstrand, MB Russell, M Leone, SL John, RC Trembath

**Affiliations:** 1 Department of Medical & Molecular Genetics, King’s College London, Guy’s Hospital, London, UK; 2 Pfizer, Neusentis, PTx Precision Medicine, Granta Park, Cambridge, UK; 3 Pfizer, PTx Precision Medicine, Eastern Point Road, Groton CT, USA; 4 Pain & Sensory Disorders, Pfizer, Neusentis, Granta Park, Cambridge, UK; 5 Department of Neurology, Karolinska University Hospital Huddinge, Karolinska Institute, Stockholm, Sweden; 6 Head and Neck Research Group, Research Centre, Akershus University Hospital, Oslo, Norway; 7 Carlo Besta National Neurological Institute, Milan, Italy

## Introduction

A genetic predisposition to cluster headache (CH) has long been debated. Genetic epidemiological studies have reported an increased risk of CH in relatives of CH sufferers. The familial clustering supports a model of autosomal dominant inheritance with reduced penetrance. Some candidate gene studies have been performed, detecting associations with HCRTR2 and ADH4, but these have been limited by small sample size. We have established a large cohort of CH families in which we have previously reported a genome-wide linkage scan, isolating a number of putative linkage loci[1]. Despite this, a single causative gene is yet to be identified, largely due to substantial genetic heterogeneity.

## Purpose

To further delineate the genetic architecture underlying CH, we have used an exome sequencing strategy in a subset of Northern European families.

## Methods and results

Exome target enrichment and paired-end sequencing were performed for ten probands. Annotated variants were filtered to exclude known polymorphisms, leaving a total of 1711 novel variants. Exome data from related affected subjects were examined to limit the analysis to variants segregating with the CH phenotype. Segregation analysis by Sanger sequencing in all family members reduced the candidate list to a total of 45 genes (range: 1-13 genes per pedigree). These genes are now being screened in our extended cohort to provide further insight into the role of each gene in CH pathogenesis.

## Conclusion

Whilst exome sequencing for rare monogenic disorders is now well-established, approaches to detect pathogenic variation with complexities such as locus heterogeneity and incomplete penetrance remain challenging. We have combined exome and Sanger sequencing to isolate novel coding variation segregating across CH families, highlighting the need for large homogeneous cohorts to elucidate the molecular genetic basis of CH. The significance of these variants in CH pathogenesis remains to be determined; however these results provide further evidence for a potential genetic predisposition to this debilitating disorder.
